# Prolonged luteal phase support with progesterone may increase papules and plaques of pregnancy frequency in pregnancies through *in vitro* fertilization^☆^^☆☆^

**DOI:** 10.1016/j.abd.2020.09.002

**Published:** 2021-02-02

**Authors:** Nur Dokuzeylul Gungor, Tugba Gurbuz, Tugba Ture

**Affiliations:** a Department of Reproductive Medicine and IVF, Goztepe Medical Park Hospital, Istanbul, Turkey; b Department of Obstetrics and Gynecology, Nisantasi University and Private Medistate Hospital, Istanbul, Turkey; c Department of Dermatology, Private Clinic, Istanbul, Turkey

**Keywords:** IVF, Plaques of pregnancy, Pregnancy, Pruritic urticarial papules

## Abstract

**Background:**

Pruritic urticarial papules and plaques of pregnancy development may have a strong relationship with hormone treatments during in vitro fertilization and hormonal changes during pregnancy.

**Objectives:**

The aim of this study was to evaluate and compare the frequency of papules and plaques of pregnancy and related factors in in vitro fertilization pregnancies and spontaneous pregnancies.

**Methods:**

In this study, 517 in vitro fertilization pregnancies and 1253 spontaneous pregnancies were retrospectively reviewed for papules and plaques of pregnancy frequency. The diagnosis of papules and plaques of pregnancy was performed by referral to the dermatology department and according to the typical clinical manifestations of the disease.

**Results:**

The papules and plaques of pregnancy was more common in all in vitro fertilization pregnancies (including single pregnancies) than in spontaneous pregnancies. Age, Rh positivity, mother weight gain, onset of disease during gestation, duration of disease, birth weight and the frequency of male fetus were similar between the two groups (p > 0.05). The rate of multiple pregnancies was higher in in vitro fertilization pregnancies with papules and plaques of pregnancy than in vitro fertilization pregnancies without papules and plaques of pregnancy (p < 0.001). Duration of progesterone treatment was also significantly longer in in vitro fertilization pregnancies with papules and plaques of pregnancy compared to in vitro fertilization pregnancies without papules and plaques of pregnancy (p < 0.001).

**Study limitations:**

The limitations of the study were the retrospective and single-centered design.

**Conclusion:**

The results of this study indicate that increased progesterone dosage or prolonged treatment may play a role in the pathogenesis papules and plaques of pregnancy.

## Introduction

The condition defined as pruritic urticarial papules and plaques of pregnancy (PUPPP), which is also known as polymorphic eruption of pregnancy, is a benign, self-limiting skin disorder that is associated with pregnancy.^1^, ^2^ It was first described by Lawley in 1979.^3^ The reported incidence of PUPPP is 0.5% in single pregnancies, 2.9% to 16% in twin pregnancies, and 14% to 17% in triplet pregnancies.^4^, ^5^ It is more prevalent in primiparous women.^6^ Although the etiopathogenesis of PUPPP has not been clearly understood, various factors, including multiple pregnancy, excessive maternal weight gain, male fetus, Rh factor presence/absence, increased progesterone receptor immunoreactivity, and high progesterone levels, are considered to play a combined role.^7^, ^8^, ^9^, ^10^ Patients with PUPPP usually experience the onset of papules and plaques in the lower abdomen and the upper regions of the lower extremities during the third trimester.^11^ Although pruritis is common and bothersome, systemic problems are not encountered in this condition, and existing symptoms almost always regress swiftly after birth.^12^

Despite the high frequency of the disease and some large studies, there is little evidence to suggest a direct relationship with any clinical or laboratory parameters.^11^ However, previous studies focused on this topic have consistently shown high progesterone levels, multiple pregnancies, and immunoreactivity of the progesterone receptor in patients who develop PUPPP.^13^, ^14^ Considering this hormonal relationship and the fact that Ghazeeri et al. reported a very high incidence of PUPPP in *in vitro* fertilization (IVF) pregnancies, it appears that PUPPP development may have a strong relationship with hormone treatments during IVF and hormonal changes during pregnancy.^10^

Therefore, this study aimed to assess PUPPP frequency in IVF patients and spontaneous pregnancies at this center, and to determine any factors associated with PUPPP onset.

## Methods

In this study, 517 IVF pregnancies and 1,253 spontaneous pregnancies followed between 2014 and 2020 were retrospectively reviewed for PUPPP diagnosis. All PUPPP diagnoses were made by experienced dermatologists according to the typical clinical manifestations of the disease. After reviewing all patients, pregnant women with known chronic illnesses and those who had been diagnosed with any cutaneous diseases before the pregnancy were excluded. The frequency of PUPPP was evaluated for the overall population in both groups, and its frequency among those with single pregnancies was also calculated. Patients in the spontaneous pregnancy group and in the IVF pregnancy group were compared in terms of the following parameters: age of gestation, multiple pregnancy, Rh positivity, mother weight gain, onset of PUPP at gestation, duration of PUPP, delivery time, caesarean section rate, infant’s birth weight, and frequency of male newborn. IVF pregnancies with and without PUPPP were also compared in terms of progesterone support treatments, in addition to the aforementioned parameters.

This study was approved by the ethical review board of Maltepe University Clinical Research Ethical Committee. Informed consent was obtained from all individual participants included in the study. SPSS (v. 22.0) for Windows was used for the statistical analysis of data. Descriptive statistics were given as frequency and percentages for categorical variables, and as mean, standard deviation, minimum, maximum, and median values for numerical variables. The statistical alpha significance level was accepted as p < 0.05.

## Results

While eight of the PUPPP cases in the spontaneous group were primigravid and three were multiparous, all patients with PUPPP in the IVF group were primigravid or nulliparous.

In overall comparisons, PUPPP was observed in 7.4% (38/517) of IVF and in 0.9% (11/1253) of spontaneous pregnancies (p = 0.006). When single pregnancies in these groups were compared, PUPPP frequency was 2.3% (10/362) in IVF *vs.*0.6% (7/1206) in spontaneous pregnancies (p < 0.001; Table 1).

**Table 1:** Comparison of PUPPP frequency in all pregnancies and single pregnancies regarding type of conception.

PUPPP frequency in all pregnancies, n (%)	IVF pregnancies (n = 517)	Spontaneous pregnancies (n = 1,253)	p
	7.4 (38 of 517)	0.9 (11 of 1,253)	0.006
PUPPP frequency in single pregnancies, n (%)	IVF pregnancies (n = 362)	Spontaneous pregnancies (n = 1206)	p
	2.3 (10 of 362)	0.6 (7 of 1,206)	< 0.001

p< 0.05, statistically significant significant p-values are shown in bold.

NS, not significant; IVF, *in vitro* fertilization; PUPPP, pruritic urticarial papules and plaques of pregnancy.

The comparison of PUPPP cases with regard to type of pregnancy yielded the following results: multiple pregnancy rate and caesarean delivery rates were significantly higher in IVF pregnancies with PUPPP when compared with spontaneous pregnancies with PUPPP (p = 0.022 and p = 0.038, respectively). Age, Rh positivity, mother weight gain, onset of PUPPP at gestation, duration of PUPPP, and birth weight of the baby and the frequency of male newborns were similar between the two groups (Table 2).

**Table 2:** Comparison of PUPPP cases in IVF and spontaneous pregnancies.

	PUPPP in IVF pregnancies (n = 38, fetus n = 66)	PUPPP in spontaneous pregnancies (n = 11, fetus n = 15)	p
Age of gestation (years), mean ± SD (range)	29.1 ± 4.4 (20–40)	29.9 ± 3.8 (20–40)	0.588
Multiple pregnancy rate (%)	73.7 (28/38)	36.4 (4/11)	0.022
Rh positivity (%)	89.4 (59/66)	86.7 (13/15)	0.762
Mother weight gain (kg), mean ± SD	17.4 ± 4.1	17.0 ± 3.9	0.847
Frequency of GDM (%)	7.9 (3/38)	0 (0/11)	0.336
Onset of PUPPP at gestation (weeks), mean ± SD	31.6 ± 1.7	32.7 ± 1.8	0.092
Duration of PUPPP (days), mean ± SD	41.6 ± 8.5	40.5 ± 5.7	0.688
Delivery time (weeks), mean ± SD	36.5 ± 1.5	37.1 ± 1.9	0.269
Caesarean delivery (%)	84 (32/38)	55 (6/11)	0.038
Birth weight of baby (grams), mean ± SD	3,063 ± 448	3,051 ± 608	0.927
Male newborn (%)	68.2 (45/66)	80(12/15)	0.366

Significant p-values are shown in bold.

IVF, *in vitro* fertilization; PUPPP, pruritic urticarial papules and plaques of pregnancy; NS, not significant; GDM, gestational diabetes mellitus.

IVF pregnancies with and without PUPPP were compared; multiple pregnancy frequency and the duration of progesterone support were significantly higher in IVF patients with PUPPP when compared with IVF patients without PUPPP (p < 0.001 and p < 0.001, respectively). Age, Rh positivity, mother weight gain, onset of PUPPP at gestation, duration of PUPPP, caesarean section rate, birth weight of the baby, and the frequency of male newborns were similar between the two groups (Table 3).

**Table 3:** Comparison of IVF pregnancies with and without PUPPP regarding possible clinical parameters.

	IVF pregnancies with PUPPP (n = 38)	IVF pregnancies without PUPPP (n = 479)	p
Age of gestation (years) mean ± SD	29.1 ± 4.4	29.3 ± 3.5	0.740
Multiple pregnancy rate (%)	73.7 (28/38)	26.5 (127/479)	<0.001
Rh positivity (%)	89.4 (59/66)	88.6 (537/606)	0.849
Mother weight gain (kg) mean ± SD	17.4 ± 4.1	17.2 ± 4.3	0.723
Frequency of GDM, n (%)	7.8 (3/38)	5.8 (28/479)	0.490
Onset of PUPP at gestation (weeks) mean ± SD	31.4 ± 1.6	32.5 ± 1.7	0.419
Duration of PUPPP (days) mean ± SD	36.4 ± 1.6	37.2 ± 1.7	0,097
Delivery time (weeks) mean ± SD	36.5 ± 1.5	36.3 ± 1.2	0.425
Caesarean delivery (%)	84 (32/38)	80 (380/479)	0.472
Birth weight of baby (grams), mean ± SD	3,063 ± 448	2,980 ± 428	0.321
Male newborn (%)	68.2 (45/66)	61.2 (371/606)	0.269
Duration of progesterone support (days), mean ± SD (range)	101 ± 35	76 ± 12	< 0.001

Significant p-values are shown in bold.

IVF, *in vitro* fertilization; PUPPP, pruritic urticarial papules and plaques of pregnancy; GDM, gestational diabetes mellitus;

## Discussion

The primary finding of this study was that PUPPP frequency was significantly higher in IVF pregnancies than in spontaneous pregnancies, regardless of the presence of single or multiple pregnancy. To the best of the authors’ knowledge, this is the first study to compare PUPPP frequency in IVF pregnancies and spontaneous pregnancies while taking into account various characteristics of patients. Only one previous study in the literature reported the higher frequency of PUPPP in IVF pregnancies.^10^ The high frequency of multiple fetuses in IVF pregnancies may be one of the factors that contribute to increased PUPPP frequency in these patients.^4^, ^5^ Multiple pregnancy causing excessive abdominal distension is suggested as the most common factor which could trigger PUPPP.^5^ This is because the eruption of lesions has been associated with damage to the connective tissue in the striae in up to 90% of patients with PUPPP.^8^ Interestingly, in the present study, abdominal circumferences were similar between IVF pregnancies and spontaneous pregnancies. Furthermore, in single IVF pregnancies, the risk for PUPPP was found to be significantly higher than in spontaneous pregnancies.

Although, PUPPP was described over four decades ago and is one of the most common dermatoses that develop during pregnancy, very little is known about its etiology. A high percentage of women with PUPPP are primigravidae. In a previous review evaluating this topic, it was reported that 42% of patients were primigravid and 68% were nulliparous.^11^ In turn, other studies put forth somewhat higher frequencies of around 70%–75%.^4^, ^7^ In the present study, all patients with PUPPP in the IVF group were primigravid or nulliparous, as they were receiving treatment because of infertility. A prospective study observing women with multiple dermatoses of the pregnancy determined an increased prevalence in women carrying male fetuses compared to female fetuses, with a ratio of 2:1.^14^ Similarly, an overall increase among those with male fetuses was observed in the present study, without statistical significance – a common situation in much of the literature. Also, in the present study, caesarean delivery rates were higher, similar to the literature.^4^, ^10^, ^15^ However, it is quite possible to attribute this relationship with the fact that caesarean delivery is utilized much more frequently in those with multiple gestation (also frequent in IVF).

In this study, an increased PUPPP incidence was observed in IVF pregnancies. To explain the cause of this increase, the clinical parameters in Table 3 were evaluated. As expected, a positive relationship was observed between PUPPP frequency and increased multiple pregnancy rates in IVF pregnancies. Perhaps more interestingly, the duration of progesterone support was also found to be longer in IVF pregnancies with PUPPP when compared with IVF pregnancies without PUPPP. In IVF, progesterone support is applied in a standard fashion and similar doses are given in the majority of patients. This result suggests that the increased cumulative dosage of progesterone in IVF patients with PUPPP may be responsible for an increased risk for PUPPP development. To the best of the authors’ knowledge, this is the first time that prolonged progesterone treatment in IVF pregnancies has been associated with PUPPP. Previous studies that discussed a possible relationship between higher progesterone and PUPPP, most commonly focused on patients that had higher levels of progesterone in association with spontaneous multiple pregnancies.^14^ In terms of clinical outcomes and suggestions, the present findings indicate that clinicians and patients should be aware of increased PUPPP risk in IVF pregnancies, especially in the presence of a need for prolonged progesterone support.

Similar to the previous report of Ghazeeri et al., the present study also revealed a higher Rh positivity percentage in patients with PUPPP.^10^ Except for this study, there are no reports supporting this result in the literature. In order to clarify the etiopathogenesis of PUPPP, the authors believed it would be useful to examine Rh positivity in more detail. In agreement with the literature, excessive maternal weight gain (> 15 kg) was also detected in the present patients with PUPPP.^4^, ^9^ Excessive weight gain increases the frequency of PUPPP by increasing abdominal skin distension. Cohen et al. found that weight gain and twin pregnancy frequency were higher in pregnant women with PUPPP.^15^ It has been suggested that a possible reason for this positive relationship between weight gain and PUPPP may be that collagen antigens resulting from abdominal distension trigger the inflammatory reaction observed in PUPPP cases.^16^ This hypothesis is also supported by studies reporting that PUPPP is observed in an earlier phase in multiple pregnancies.^17^, ^18^ Upon a strong suggestion of PUPPP diagnosis, the degree of discomfort related to pruritus should be evaluated on a patient-by-patient basis, as the treatment is mainly symptomatic. All patients the present study were treated symptomatically with moisturizing creams and topical corticosteroids. Cool, soothing baths, application of emollients, and the use of light cotton clothing were also suggested to patients and additional symptomatic relief was reported, similarly to the literature.^4^, ^9^ Even though PUPPP regresses after delivery, it is definitely not an indication for early delivery, due to the fact that PUPPP is almost always self-limiting and benign. Therefore, none of the patients in this study underwent early birth due to the presence of PUPPP.^19^, ^20^ It is a general consensus that PUPPP poses a negligible risk to the mother or fetus; however, some degrees of adverse relationships have been reported in some cases. For instance, a case report showed a relationship with hypertension and mild preeclampsia and induction of early labor in association with hypertension development.^21^ Although these problems are relatively rare, they should still be kept in mind for patients with PUPPP. In the current study, three patients had gestational diabetes in the IVF pregnancy group.

The most important limitation of this study is the small percentage of patients with PUPPP relative to the very large size of both study groups. However, the number of patients with PUPPP included in this study was considerably higher when compared with the majority of studies in the literature. Moreover, this is a retrospective analysis of patients which would limit the objective characterization of patients and could induce biases in patient assessment. Finally, this is a single center study, but its strength lies in its detailed analysis of meticulously kept medical records.

## Conclusions

In this study, PUPPP was found to be statistically more frequent in IVF pregnancies when compared with spontaneous pregnancies, and the authors also found that duration of progesterone treatment was associated with PUPPP. These results indicate that cumulative dosage of progesterone or prolonged treatments may play a role in the pathogenesis of PUPPP. Further studies involving a larger number of patients representing different communities should be performed to confirm our findings and determine variations within/between different populations. The role of progesterone in the onset of PUPPP should also be identified through *in vitro* and *in vivo* studies.

## Financial support

None declared.

## Authors' contributions

Nur Dokuzeylul Gungor: Approval of the final version of the manuscript; preparation and writing of the manuscript; study conception and planning; effective participation in research orientation; intellectual participation in propaedeutic and/or therapeutic management of studied cases; manuscript critical review; critical literature review.

Tugba Gurbuz: Statistical analysis; critical literature review.

Tugba Ture: Data collection, analysis, and interpretation; study conception and planning; effective participation in research orientation; intellectual participation in propaedeutic and/or therapeutic management of studied cases.

## Conflicts of interest

None declared.
